# Transcriptome Analysis of Solute Carrier-Associated Genes in Hepatocellular Carcinoma: Friend or Foe?

**DOI:** 10.3389/fgene.2022.856393

**Published:** 2022-03-23

**Authors:** Wei Wei, Rubin Xu, Xiaomei Ying, Liang Chen, Xiaohuan Lu, Qikai Tang, Jiaheng Xie, Hongzhu Yu

**Affiliations:** ^1^ Department of General Surgery, Fuyang Hospital of Anhui Medical University, Fuyang, China; ^2^ Department of General Surgery, Suzhou Hospital of Anhui Medical University, Suzhou, China; ^3^ Department of Gastrointestinal Surgery, Union Hospital, Tongji Medical College, Huazhong University of Science and Technology, Wuhan, China; ^4^ Department of Neurosurgery, The First Affiliated Hospital of Nanjing Medical University, Jiangsu Province Hospital, Nanjing, China; ^5^ Department of Burn and Plastic Surgery, The First Affiliated Hospital of Nanjing Medical University, Jiangsu Province Hospital, Nanjing, China

**Keywords:** hepatocellular carcinoma, signature, solute carrier, transporter, bioinformatics analysis

## Abstract

Hepatocellular carcinoma (HCC) is one of the most common types of cancer, and its treatment remains difficult. Since the early symptoms of HCC are not obvious, many HCC patients are already at an advanced stage of the disease at the time of diagnosis. Although current targeted therapy and immunotherapy have been initially effective in HCC patients, several patients have shown low response rates or developed drug resistance, which leads to tumor progression and even death. Hence, there is an urgent need for new biomarkers to guide the prognosis and treatment of HCC. In our study, a prognostic signature consisting of nine SLC genes was constructed in HCC by comprehensive analysis. By calculating risk scores, HCC patients could be divided into high-risk and low-risk groups, with the high-risk group having a significantly poorer prognosis. In addition, we found a hub gene, SLC7A11, which is a robust prognostic marker of HCC. In conclusion, our study can serve as a reference for the prognostic evaluation and treatment of HCC.

## Introduction

Hepatocellular carcinoma (HCC), the most common subtype of liver cancer, is currently one of the three leading causes of cancer-related deaths worldwide (Hartke et al., 2017). Overall, global incidence of HCC is continuing to increase (Sim and Knox, 2018). Due to the absence of early symptoms and signs, many HCC patients have already experienced tumor spread before receiving a definitive diagnosis (Degasperi and Colombo, 2016). For advanced HCC, the FDA-approved first-line drug sorafenib is a targeted therapy (Xia et al., 2020). Although sorafenib can prolong the survival of patients with advanced HCC by several months, these few months are of little significance in terms of achieving a cure, and the majority of patients still die (He et al., 2019). There is an urgent need for new biomarkers to guide the prognosis of HCC patients and for therapeutic exploration to be conducted on this basis (Hartke et al., 2017). As the liver is the largest metabolic and immune organ in the human body, its physiological process requires the participation of many transmembrane transporters (Jia et al., 2018). Moreover, the carcinogenesis of hepatocytes will inevitably involve changes in transmembrane transporters (Fan et al., 2018). It is of great value to explore the role of transmembrane transporters in hepatocellular carcinoma.

The solute carrier (SLC) transporter family is one of the most important material transporters on the human cell membrane (Pizzagalli et al., 2021). Their substrates are extensive and include sugars, vitamins, nucleotides, amino acids, ions, and drugs, among others (Wu et al., 2021). Unlike ATP-binding cassette (ABC) transporters, SLC transporters are typically not powered by direct hydrolysis of ATP but are active secondary transporters which operate via existing concentration gradients (Marin et al., 2020). They are closely related to cellular homeostasis and many pathophysiological processes. At present, there are a number of studies which have preliminarily explained the significance of SLC transporters in HCC. Gao et al. found that SLC27A5 is a novel tumor suppressor in HCC (Gao et al., 2020). He et al. found that the upregulation of SLC7A11 mediated HCC metastasis in the context of inflammation. Thus, the role of the SLC family in HCC is potentially significant (He et al., 2021).

The development of bioinformatics and related databases has made the exploration of tumor genomics more convenient and accurate (Li et al., 2021). Among these databases, the TCGA and ICGC databases are the two most commonly used, since they contain a large amount of high-throughput sequencing data and related clinical information (Cao et al., 2021). By analysing these high-throughput sequencing data, we can discover many novel biomarkers to guide the diagnosis and treatment of diseases.

In this study, we comprehensively analysed all members of the SLC family in HCC and constructed the prognostic signature by Cox regression and Least Absolute Shrinkage and Selection Operator (LASSO) regression. By calculating their risk scores, we divided HCC patients into high-risk and low-risk groups; differences could be observed between the two groups in terms of prognosis and immune cell infiltration. Overall, our study provides a reference for the prognostic assessment and treatment of HCC.

## Methods

### Download of Data

Using the UCSC Xena tool (https://portal.gdc.cancer.gov/), gene expression data and clinicopathological data of HCC samples were obtained from the TCGA database (comprising 370 tumor samples and 50 normal samples) for use as the training cohort. 240 HCC samples including expression data and clinicopathological data were used as the validation cohort, these being obtained from the ICGC database (http://dcc.icgc.org/). A total of 456 SLC-related genes were obtained from the bioparadigms database (http://slc.bioparadigms.org/). The clinical information of the two cohorts is summarized in Table 1.

**TABLE 1:** Clinical information of the two cohorts.

Characteristic	The Clinicopathological features of TCGA			The Clinicopathological features of ICGC	
		Samples (N = 350)	Percentage (%)	Samples (N = 240)	Percentage (%)
Fustat					
	0	233	67%	197	82.08%
	1	117	33%	43	17.92%
Age					
	≤65	227	65%	92	38.33%
	>65	123	35%	148	61.67%
Gender					
	Female	110	31%	61	25.42%
	Male	240	69%	179	74.58%
Stage					
	Stage I	174	50%	36	15.00%
	Stage II	85	24%	109	45.42%
	Stage III	86	25%	74	30.83%
	Stage IV	5	1%	21	8.75%
Grade					
	G1	45	13%		
	G2	172	49%		
	G3	120	34%		
	G4	13	4%		
T					
	T1	176	50%		
	T2	88	25%		
	T3	76	22%		
	T4	10	3		

### Screening of SLC Genes With Differential Expression and Prognostic Value in HCC

R software (4.1.2) was used for screening and survival analysis of SLC genes differentially expressed between normal tissues and HCC. The “limma” R package was used for difference analysis, and the SLC-related genes showing differences were screened by setting logFCfilter = 0.5 and fdrFilter = 0.05 as truncation values. The “pheatmap” and “ggplot2” R packages were used for visual mapping of heat maps and volcano maps, respectively. The Metascape website (http://metascape.org) was used for pathway enrichment analysis. Differentially expressed SLC genes in the TCGA and ICGC cohorts were obtained by difference analysis. Then, the most significantly differentially expressed SLC gene was obtained from the intersection between the two cohorts. Subsequently, univariate Cox regression was performed to screen SLC genes associated with survival (Table 2). The “survival” R package was used for Cox regression analysis and the *p*-value was set to 0.05 as the cut-off value.

**TABLE 2:** Univariate Cox regression was performed to screen SLC genes associated with survival.

Gene	Hr	HR.95L	HR.95H	Pvalue
SLC7A1	1.429002	1.166092	1.751188	0.000579
SLC26A6	1.39464	1.128452	1.72362	0.002082
SLC25A19	1.821355	1.358969	2.441068	6.00E-05
SLC26A2	1.632969	1.184107	2.251982	0.002786
SLC22A15	1.410603	1.143838	1.739584	0.001298
SLC2A5	1.195645	1.03568	1.380317	0.014753
SLC16A3	1.349503	1.192081	1.527714	2.18E-06
SLC13A4	4.014419	1.649087	9.772413	0.002199
SLCO2A1	0.753936	0.627799	0.905416	0.002498
SLC22A5	1.563664	1.059449	2.307847	0.024403
SLC10A4	1.80009	1.128509	2.87133	0.013609
SLC36A1	2.066032	1.491982	2.86095	1.25E-05
SLC5A11	1.2537	1.023908	1.535064	0.028621
SLC38A6	1.634001	1.255578	2.126479	0.000259
SLC17A8	5.879836	2.232647	15.48497	0.000336
SLC7A11	1.484287	1.24096	1.775326	1.54E-05
SLC7A6	1.504794	1.071163	2.11397	0.018455
SLC39A10	1.77464	1.361445	2.313239	2.22E-05
SLC6A3	1.686828	1.129537	2.519074	0.010611
SLC6A8	1.119546	1.002878	1.249786	0.04431
SLC30A3	1.301811	1.097402	1.544296	0.002474
SLC35E4	1.834531	1.274206	2.641254	0.001102
SLC47A2	2.368396	1.238203	4.530191	0.00917

### Construction of the SLC-Related Prognostic Signature in HCC

After obtaining the SLC genes with differential expression and prognostic value, we used the Least Absolute Shrinkage and Selection Operator (LASSO) regression algorithm to construct the prognostic signature for quantifying each patient’s risk score. First, HCC patients in the TCGA cohort were divided into a high-risk group and a low-risk group based on the median risk score; this was followed by survival analysis of the two groups. The “survival” and “survminer” R packages were used for Kaplan-Meier survival analysis and visualization (*p* < 0.05 was considered a significant difference). The “timeROC” R package was then used to construct ROC curves to assess the robustness of the signature. Finally, risk grouping, survival analysis and ROC curve construction in the ICGC cohort were also carried out in order to verify the accuracy and repeatability of the signature.

### Further Evaluation of the SLC-Related Signature

To evaluate the constructed signature, the following five analyses were performed in the training set and validation set: risk score, survival status, principal component analysis (PCA), t-SNE analysis, and Cox regression analysis for clinical factors. Firstly, we sorted the samples according to their risk score and survival status and used the “pheatmap” R package to draw the risk curve and survival status map. PCA and t-SNE analysis were then performed using the R software packages “Rtsne” and “ggplot2”, respectively. Finally, univariate and multivariate Cox analyses were performed for multiple clinical features and risk scores using the “survival” R package.

### Gene Set Enrichment Analysis

Tumor progression is associated with changes in multiple functions and activation of pathways. Accordingly, we performed GSEA analysis to explore the function and pathway enrichment in the high-risk group. Gene sets for enrichment analysis were obtained from the GSEA website (https://www.gsea-msigdb.org/gsea/index.jsp). The “clusterProfiler” and “enrichplot” R packages were used for GSEA analysis. Finally, five Gene Ontology (GO) functions and Kyoto Encyclopedia of Genes and Genomes (KEGG) pathways with the highest scores were displayed and visualised.

### Analysis of Immune Microenvironment

The progression of HCC is closely related to immune cell infiltration, immune function reprogramming and immune checkpoint regulation. Consequently, we analysed the immune microenvironment. Firstly, we used the ssGSEA method to calculate the immune cell scores and immune-related function scores of all HCC samples from the TCGA cohort. The “GSVA” R package was used for analysis. We then compared the difference in immunity scores between the high- and low-risk groups (*p* < 0.05 was considered statistically significant). The “limma” R package was used for difference analysis between groups, and the “ggpubr” R package was used for visualisation of immune infiltration and activation of immune functions.

### Interaction Network Analysis and Identification of Hub Gene

We analysed the interaction between genes in the signature using the STRING database (https://cn.string-db.org/). Cytoscape software was then used to search for the hub gene. Finally, we analysed the expression, prognostic value, and correlation with immune cell infiltration of the hub gene in HCC.

### Drug Sensitivity Analysis

Chemotherapy has been widely used as a treatment for HCC patients. We hope to classify patients according to the SLC-related signature and treat them with specific drugs in order to improve the survival rates or quality of life of patients. To this end, we performed drug sensitivity analysis on the genes in the signature. Gene expression data and drug correlation data were obtained from the CellMiner database (https://discover.nci.nih.gov/cellminer/home.do). The “limma” R package was used to analyse the gene expression level and drug correlation. Pearson’s correlation coefficient was used as a statistical method, and *p* < 0.05 was considered as statistically significant.

### Construction of the Nomogram

The nomogram, calibration curve and multiple ROC curves were used to evaluate the clinical significance of this signature. Firstly, we used the “regplot”, “rms” and “survival” R packages to draw the nomogram combining the clinicopathological information of patients. The survival of the HCC patient TCGA-K7-AAU7 was predicted by this nomogram. Then, the calibration curve was used to evaluate the accuracy of the nomogram’s prediction of survival for 1, 2, and 3 years. Finally, we used the “timeROC” R package to plot the ROC curve in order to evaluate the robustness of the nomogram.

### Quantitative Real-Time Polymerase Chain Reaction

Twelve patients with hepatocellular carcinoma were recruited between March 2021 and January 2022 and underwent surgery. Hepatocellular carcinoma tissue resected during the operation was set as the disease group, and adjacent 2–5 cm tissue was set as the control group. The ethics committee of Fuyang Hospital affiliated to Anhui Medical University approved the study. Total cellular RNAs of 12 pairs of clinical specimens of hepatocellular carcinoma and adjacent tissue were isolated using Trizol Reagent (Invitrogen, Carlsbad, CA, United States) according to the manufacturer’s instructions. cDNAs were reverse transcripted with PrimeScript ™ RT reagentKit (Takara, Dalian, China). qRT-PCR was implemented utilizing AceQ Universal SYBR qPCR Master Mix (Vazyme, Nanjing, China) on an ABI Stepone plus PCR system (Applied Biosystems, FosterCity, CA, United States). Relative quantification was determined using the 2^−ΔΔCt^ method. The relative expression of messenger RNA (mRNA) for each gene was normalized to the level of β-actin mRNA. The sequences of primers were listed in Supplementary Table S1. Basic informations of patients were provided in Supplementary Table S2.

### Statistical Analysis

The Wilcoxon rank-sum test was used to analyse differences between normal tissue and tumor samples. Cox analysis and the K-M curve were used for survival analysis. Correlation analysis of drug sensitivity was conducted using the Pearson correlation coefficient. The ROC curve was used to evaluate the accuracy of the signature. *p* < 0.05 was used as the test level. R software (4.1.2) was the platform for analysis.

## Results

### Screening of SLC Genes With Differential Expression and Prognostic Value in HCC

**FIGURE 1:**
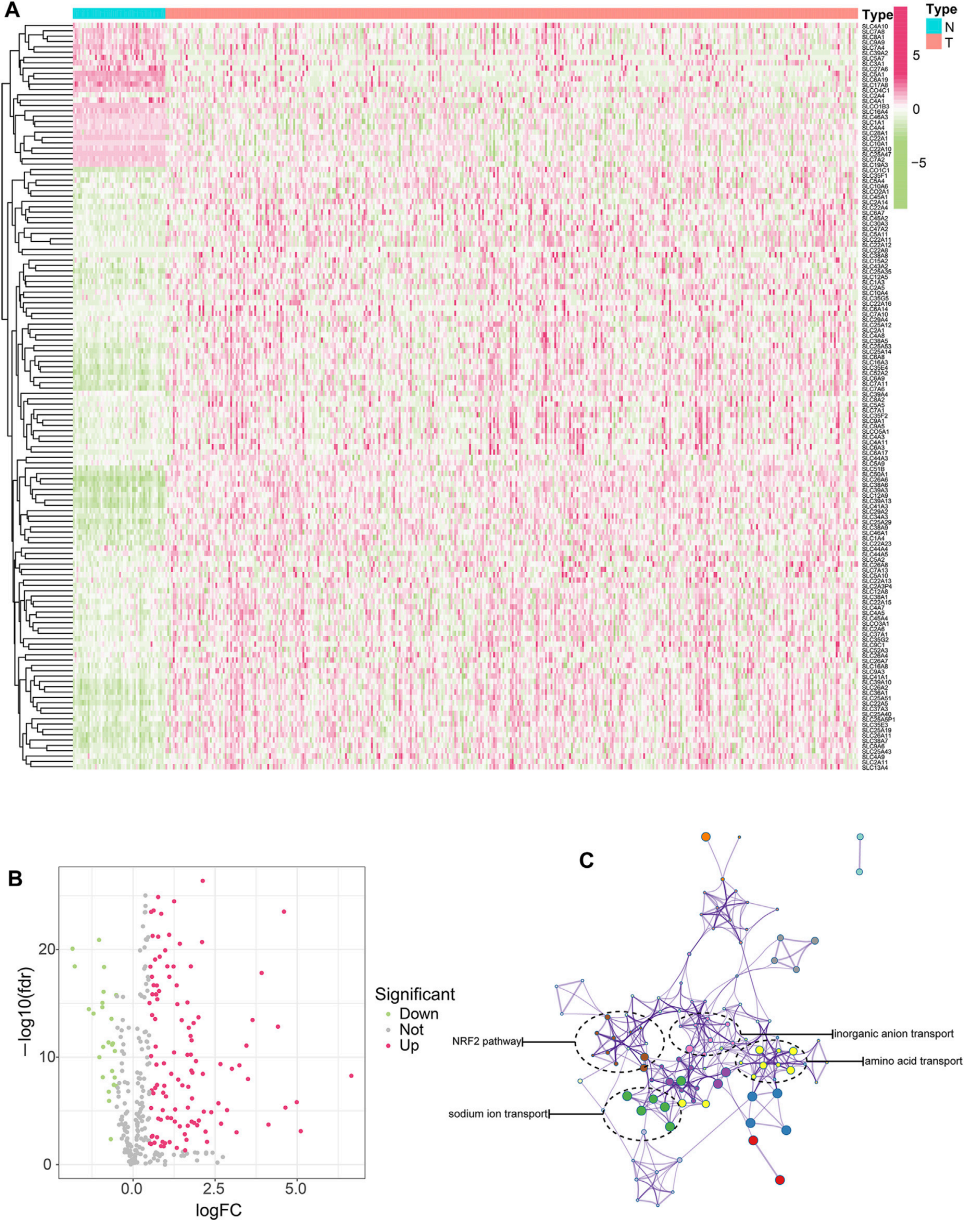
Screening of SLC genes with differential expression and prognostic value in HCC. **(A)** SLC genes differentially expressed between HCC and normal tissues were shown in heat map. Red represents high expression, green represents low expression, and the darker the color, the more significant the difference. **(B)** SLC genes differentially expressed between HCC and normal tissues were shown in volcano map. **(C)** The interaction network of differentially expressed SLC genes and the enrichment of functions and pathways.

First of all, we took the intersection of SLC genes differentially expressed between the TCGA and ICGC cohorts, ultimately obtaining 140 SLC genes. Among these, 26 SLC genes were downregulated and 114 SLC genes were upregulated. (Figures 1A,B). We then conducted protein interaction network analysis for the 140 SLC genes and found them to be associated with multiple pathways or functions (Figure 1C), including the tumor-related antioxidant pathway Nrf2. In addition, these SLC genes were found to be closely related to cell functions relating to substance transport, such as inorganic anion transport, amino acid transport and sodium transport.

### Construction of the SLC-Related Prognostic Signature in HCC

**FIGURE 2:**
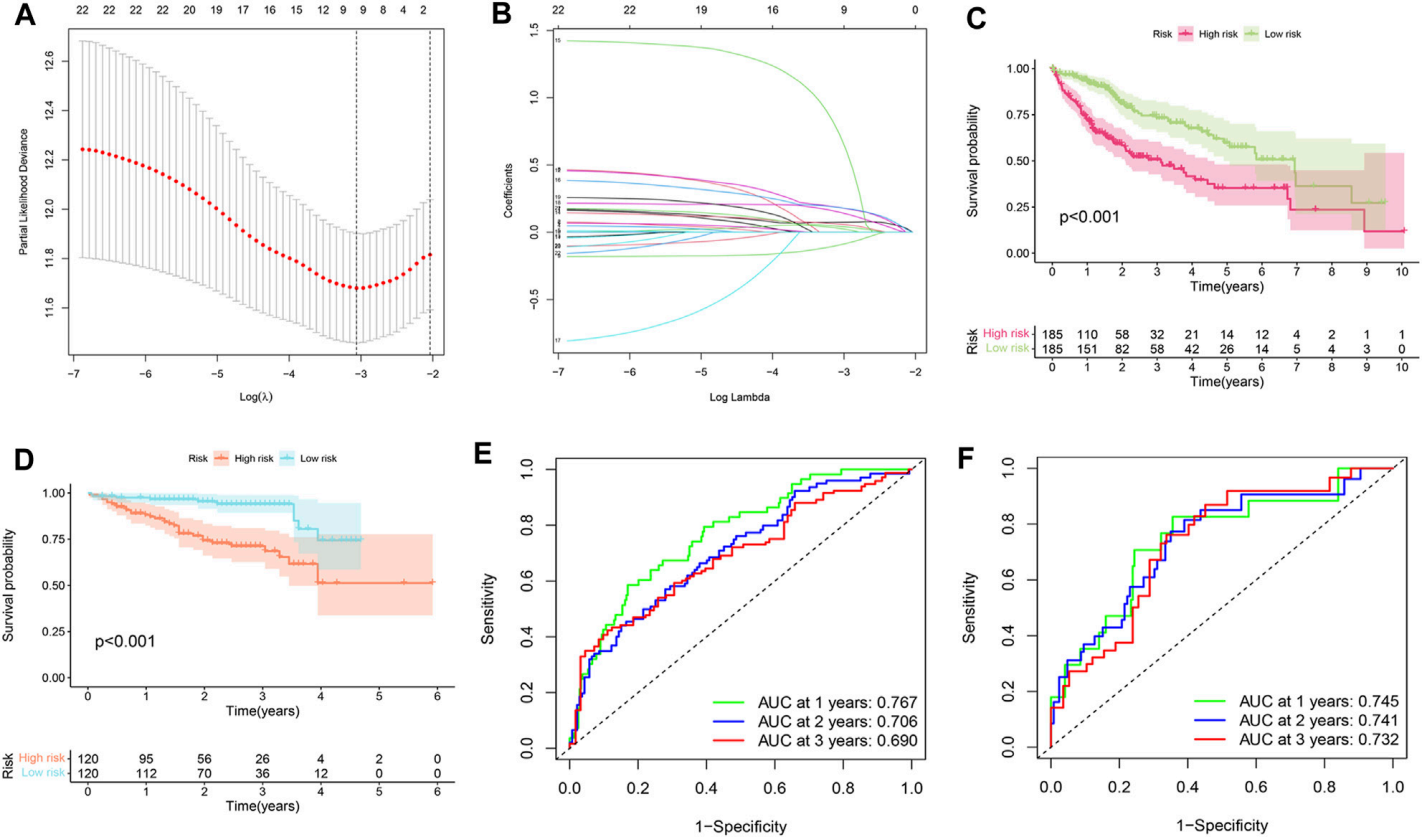
Construction of the SLC-related prognostic signature. **(A,B)** LASSO regression. The most suitable number of genes for signature construction is 9. **(C)** Survival analysis in training set (TCGA cohort). HCC patients in the TCGA cohort can be divided into high-risk and low-risk groups based on the LASSO regression risk scoring formula. The prognosis was significantly worse in the high-risk group. **(D)** Survival analysis in validation set (ICGC cohort). Similarly, patients in the high-risk group were found to have worse prognoses. **(E)** Signature’s ROC curve was built in the training set to evaluate its accuracy. The area under the curve (AUC) represents accuracy. The AUC values of 1, 2, and 3 years were 0.767, 0.706, and 0.690 respectively. **(F)** ROC curve in the validation set. The AUC values of 1, 2, and 3 years were 0.745, 0.741, and 0.732 respectively.

The SLC-related prognostic signature was constructed using the LASSO regression algorithm. As shown in Figures 2A,B, the optimal number of signature genes included is nine. They were SLC25A19, SLC16A3, SLCO2A1, SLC36A1, SLC38A6, SLC17A8, SLC7A11, SLC39A10, and SLC30A3. By attaching weights to the expression levels of these nine genes, the formula for calculating the risk score can be obtained as follows: risk score = (SLC25A19*0.043498638) + (SLC16A3*0.073392475) + (SLCO2A1*-0.094731354) + (SLC36A1*0.183471738) + (SLC38A6*0.059393544) + (SLC17A8*0.726862128) + (SLC7A11*0.183304419) + (SLC39A10*0.148495806) + (SLC30A3*0.028196825). Next, HCC patients in the TCGA and ICGC cohorts could be divided into high-risk and low-risk groups. Survival analysis showed that the prognosis of the high-risk group in the training set (TCGA cohort) and validation set (ICGC cohort) was worse than that of the low-risk group (Figures 2C,D, *p* < 0.001). In addition, ROC curves were constructed to predict the accuracy of the signature. The area under curve (AUC) values of the ROC curve of the training set at 1, 2, and 3 years were 0.767, 0.706, and 0.690, respectively (Figure 2E). The area under curve values of the ROC curve of the validation set at 1, 2, and 3 years were 0.745, 0.741, and 0.732, respectively (Figure 2F). This indicates that the signature was robust.

### Further Evaluation of the SLC-Related Signature

**FIGURE 3:**
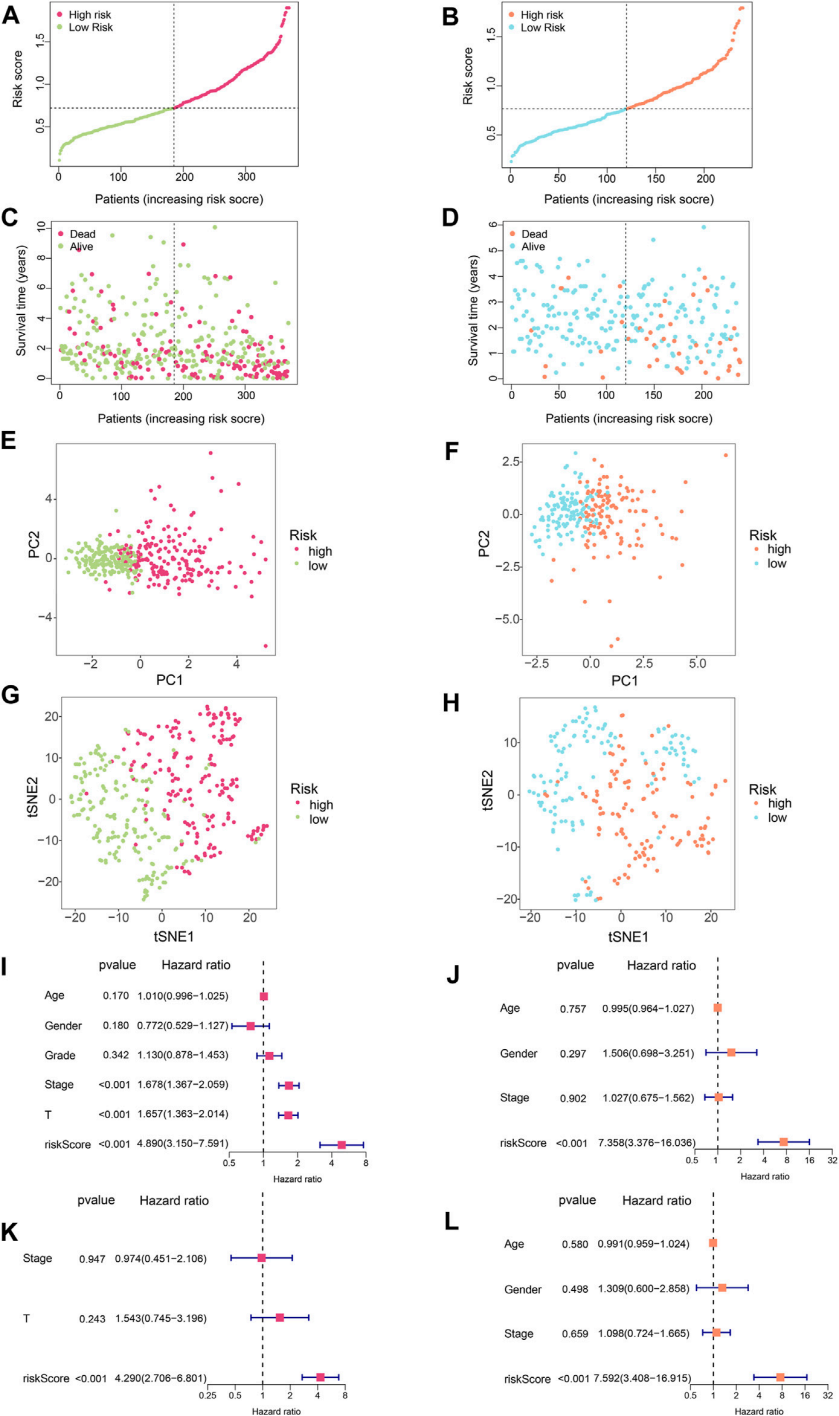
Further evaluation of the SLC-related signature. **(A)** Risk score curve of training set. **(B)** Risk score curve of validation set. **(C)** The relationship between patient survival time and risk score in the training set. The results showed that as the risk score increased, the scatter plot gradually concentrated at the bottom, meaning that the survival time became shorter. **(D)** The relationship between patient survival time and risk score in the validation set. **(E)** Principal component analysis (PCA) for training set. The results showed that HCC patients could be well divided into two groups by SLC-related signature. **(F)** Principal component analysis (PCA) for validation set. The results showed that HCC patients could be well divided into two groups by SLC-related signature. **(G)** TSNE analysis was performed on the training set. The results showed that HCC patients could be well divided into two groups by SLC-related signature. **(H)** TSNE analysis was performed on the validation set. The results showed that HCC patients could be well divided into two groups by SLC-related signature. **(I)** Univariate COX regression analysis of the training set. Stage, T-stage, and risk scores were independent prognostic factors for HCC patients (*p* < 0.001) **(J)** Univariate COX regression analysis of the validation set. Only risk score was the independent prognostic factor for HCC patients (*p* < 0.001). **(K,L)** Multivariate COX regression showed that only risk score was an independent prognostic factor in both training and validation sets (*p* < 0.001).

In order to evaluate the accuracy of this signature, we performed a series of analyses. Firstly, we visualised the risk scores of patients in the two cohorts (Figures 3A,B). Figures 3C,D show the survival states of the samples in the training set and validation set, respectively. Next, PCA and t-SNE analysis were performed, and it was shown that the HCC patients could be clearly divided into two groups according to SLC-related signature (Figures 3E–H). Finally, univariate and multivariate Cox analyses were conducted **(** Figures 3I–L). Univariate Cox regression analysis of the training set showed that stage, T-stage, and risk scores were independent prognostic factors for HCC patients (Figure 3I, *p* < 0.001). Then, in the validation set, univariate Cox regression analysis found that risk score was an independent prognostic factor for patients (Figure 3J, *p* < 0.001). Multivariate Cox regression revealed that only risk score was an independent prognostic factor in both training and validation sets. (Figures 3K–L, *p* < 0.001).

### Gene Set Enrichment Analysis

**FIGURE 4:**
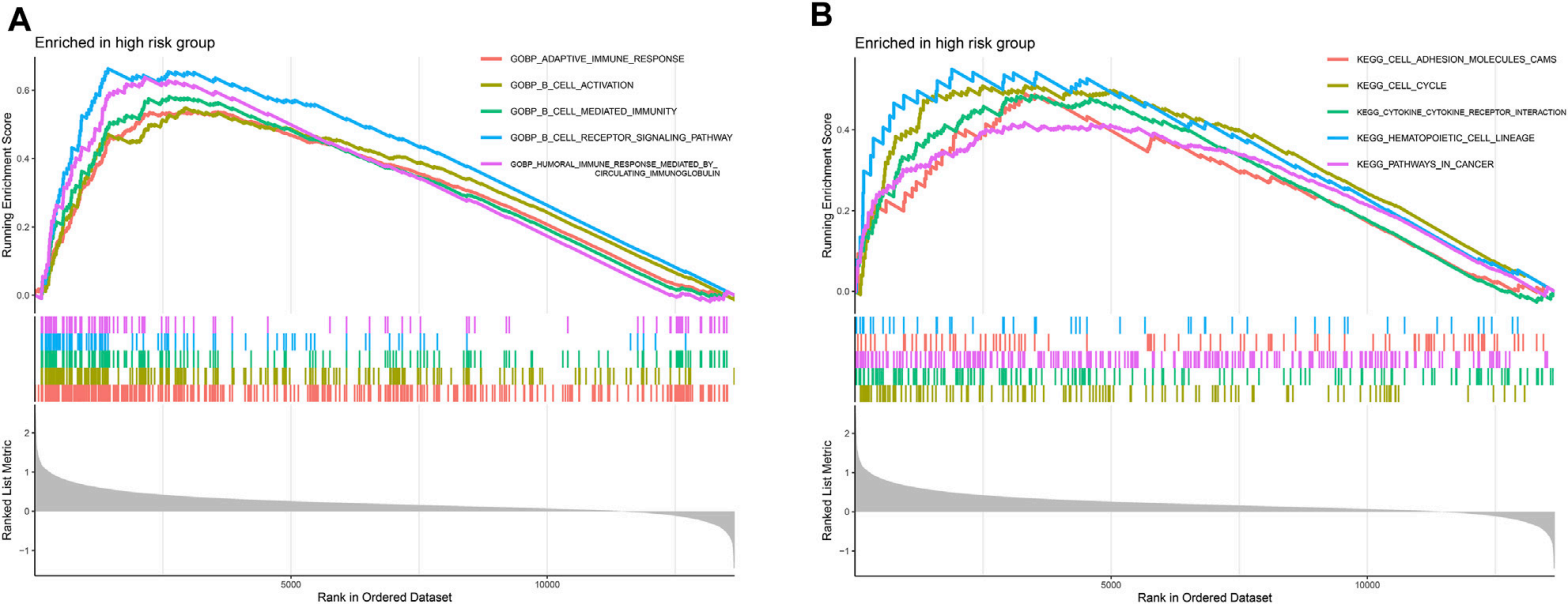
Gene set enrichment analysis (GSEA). **(A)** Functional enrichment analysis. Multiple immune functions such as adaptive immune response, B cell activation, B cell-mediated immunity, B Cell receptor signaling pathway, and humoral immune response mediated by circulating immunoglobulin were overactivated in high-risk group. **(B)** Pathway enrichment analysis. The pathways of the high-risk group were mainly enriched in cell adhesion molecules, cell cycle, cytokine receptor interaction, hematopoietic cell lineage, and pathways in cancer.

Tumor progression is associated with changes in multiple functions and activation of pathways. Since the high-risk group had a worse prognosis, we investigated functional enrichment and pathway enrichment in the high-risk group. Functional enrichment of the high-risk group was found to be strongly associated with multiple immune functions, including adaptive immune response, B cell activation, B cell-mediated immunity, B cell receptor signaling pathway, and humoral immune response mediated by circulating immunoglobulin (Figure 4A). The pathways of the high-risk group were mainly enriched in cell adhesion molecules, cell cycle, cytokine receptor interaction, hematopoietic cell lineage, and pathways in cancer (Figure 4B).

### Analysis of Immune Microenvironment

**FIGURE 5:**
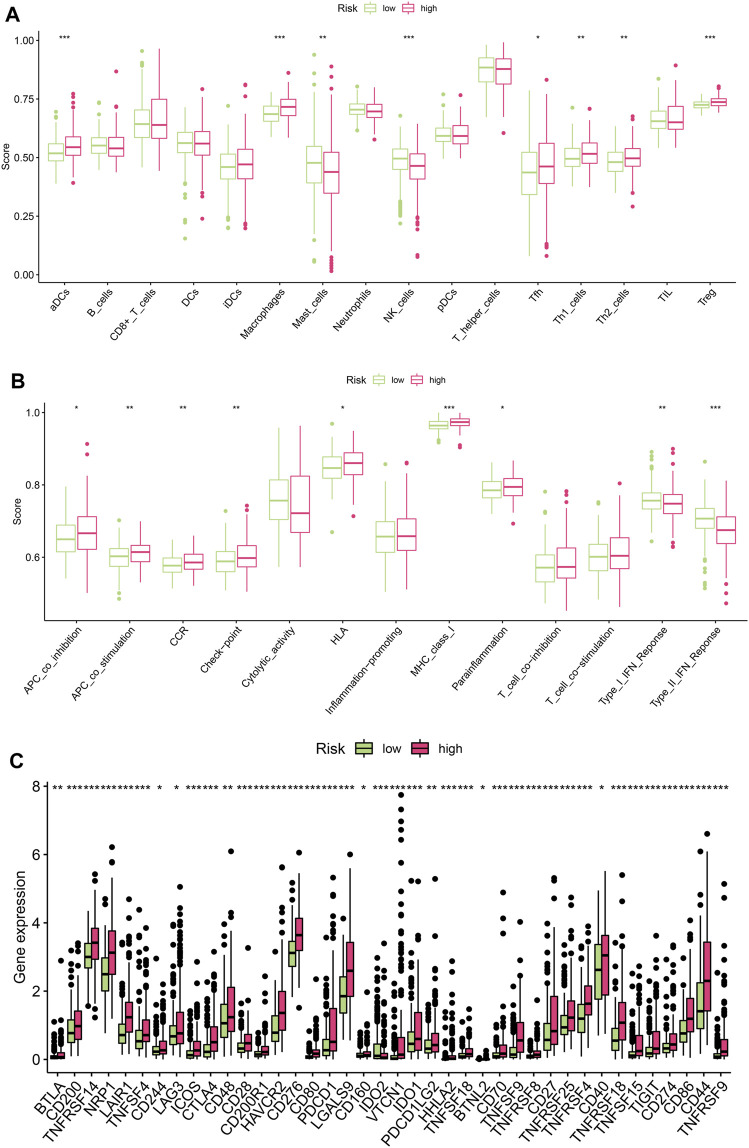
Analysis of immune microenvironment. **(A)** Differences in levels of immune cell infiltration between the high-risk and low-risk groups(*p < 0.05, **p < 0.01, ***p < 0.001) **(B)** Differences in the activation of various immune functions between the high-risk and low-risk groups (*p < 0.05, **p < 0.01, ***p < 0.001) **(C)** Differences in the expression of immune checkpoint genes between the high-risk and low-risk groups. (*p < 0.05, **p < 0.01, ***p < 0.001).

Since the above results revealed functional enrichment in the high-risk group as being related to the functions of various immune cells, the immune microenvironment was then analysed. Firstly, we analysed differences in levels of immune cell infiltration between the high-risk and low-risk groups. The results showed that for DCs, macrophages, MAST cells, NK cells, Tfh cells, Th1 cells, Th2 cells, and Treg cells, there were significant differences between the two groups in terms of infiltration levels (Figure 5A). Immune function analysis revealed differences between the two groups in terms of the activation of various immune functions, such as APC co-inhibition, APC co-stimulation, CCR, Check-point, HLA, MHC class I, parainflammation, Type-I IFN response and Type-II IFN response (Figure 5B). Finally, we analysed the differences between the two groups in terms of the expression of immune checkpoint-related genes to guide the development of immunotherapy. As shown in Figure 5C, the expression of immune checkpoint genes in the high-risk group was higher than that in the low-risk group, suggesting that the high-risk group might benefit more from immunotherapy.

### Interaction Network Analysis and Identification of Hub Gene

**FIGURE 6:**
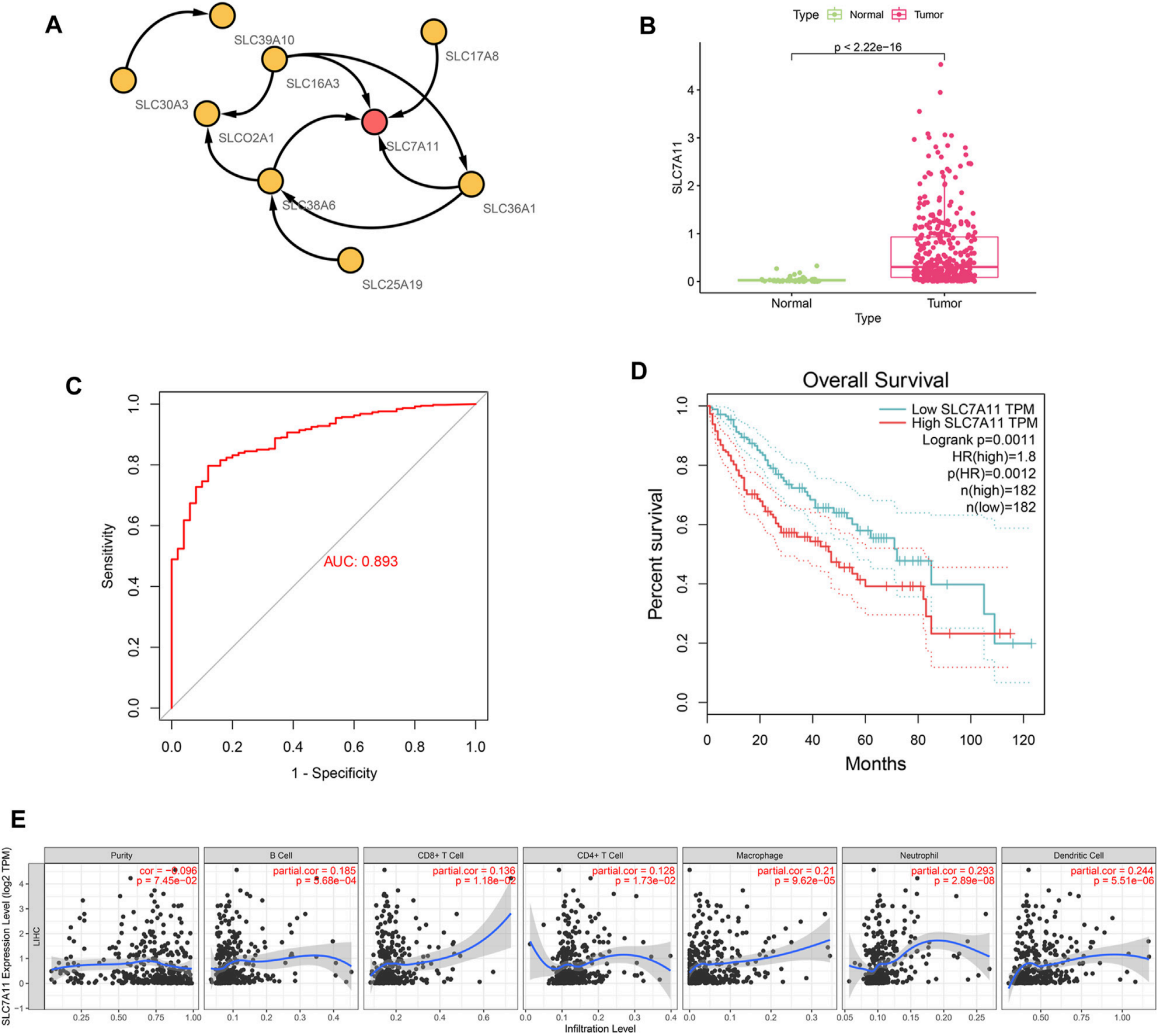
Interaction network analysis to obtain the hub gene SLC7A11. **(A)** Interaction network for 9 genes in the SLC-related signature. SLC7A11 is located in the center of the network. **(B)** SLC7A11 expression was significantly up-regulated in HCC (*p* < 2.22E-16). **(C)** SLC7A11 has strong diagnostic accuracy for HCC, and its AUC value of ROC curve is 0.893. **(D)** Survival analysis showed that upregulation of SLC7A11 was associated with poor prognosis of HCC (*p* < 0.01). **(E)** Immune infiltration analysis. The expression of SLC7A11 in HCC was positively correlated with B cell, CD8^+^ T cell, CD4^+^ T cell, macrophage, neutrophil, and dendritic cell (*p* < 0.05).

Cytoscape software was used to construct an interaction network for nine genes in the SLC-related signature and to screen hub genes. Figure 6A shows the interaction between nine signature genes, among which SLC7A11 was located at the center of the network and was the hub gene. We then performed expression analysis, ROC curve evaluation, survival analysis and immune analysis of SLC7A11 in HCC. The results showed that SLC7A11 was highly expressed in HCC samples in the TCGA database (Figure 6B, *p* < 0.001). An ROC curve was used to evaluate the accuracy of the TCGA database in diagnosing HCC, and its AUC value was 0.893. This indicates that SLC7A11 has high diagnostic value for HCC (Figure 6C). Survival analysis showed that in HCC, high expression of SLC7A11 was associated with a poor prognosis (Figure 6D, *p* = 0.0012). The results of immune infiltration showed that the expression of SLC7A11 in HCC was positively correlated with B cells, CD8^+^ T cells, CD4^+^ T cells, macrophages, neutrophils and dendritic cells (Figure 6E, *p* < 0.05).

### Drug Sensitivity Analysis

**FIGURE 7:**
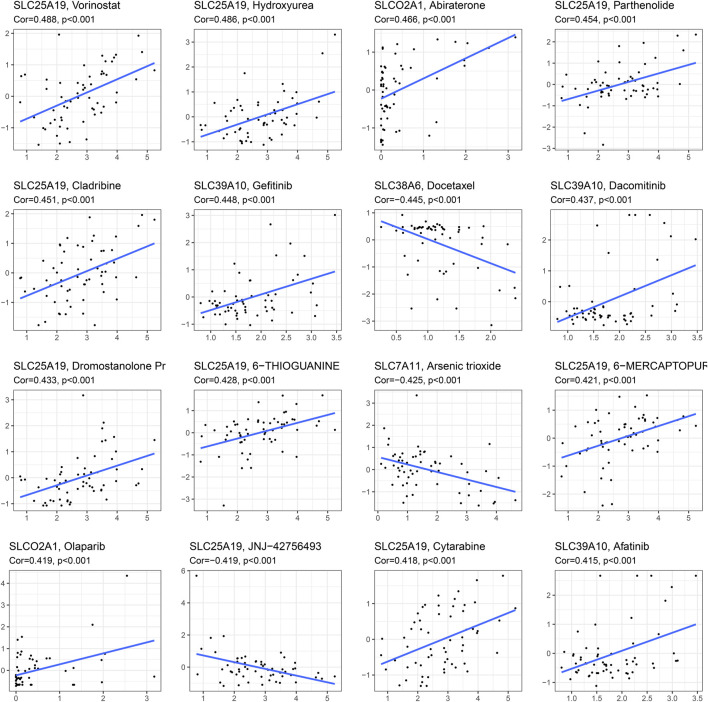
Drug sensitivity analysis. The 16 drugs most correlated with genes in the SLC-related signature (*p* < 0.001).

We performed drug sensitivity analysis on model genes to find the most effective drugs for the treatment of HCC. Figure 7 shows the 16 drugs most correlated with genes in the SLC-related signature (*p* < 0.001). We found that the hub gene SLC7A11 was negatively correlated with arsenic trioxide (Cor = −0.425). In addition, we found that SLC25A19 was significantly correlated with a variety of drugs, and was positively correlated with Vorinostat (Cor = 0.488), Hydroxyurea (Cor = 0.486), Parthenolide (Cor = 0.454), Cladribine (Cor = 0.451), Dromostanolone Pr (Cor = 0.433), 6-Thioguanine (Cor = 0.428), 6-Mercaptopurine (Cor = 0.421) and Cytarabine (Cor = 0.418). SLC25A19 was only found to be negatively correlated with JNJ−42756493 (Cor = −0.419). These results indicate that SLC25A19 may play an important role in the drug treatment of HCC.

### Construction of the Nomogram

**FIGURE 8:**
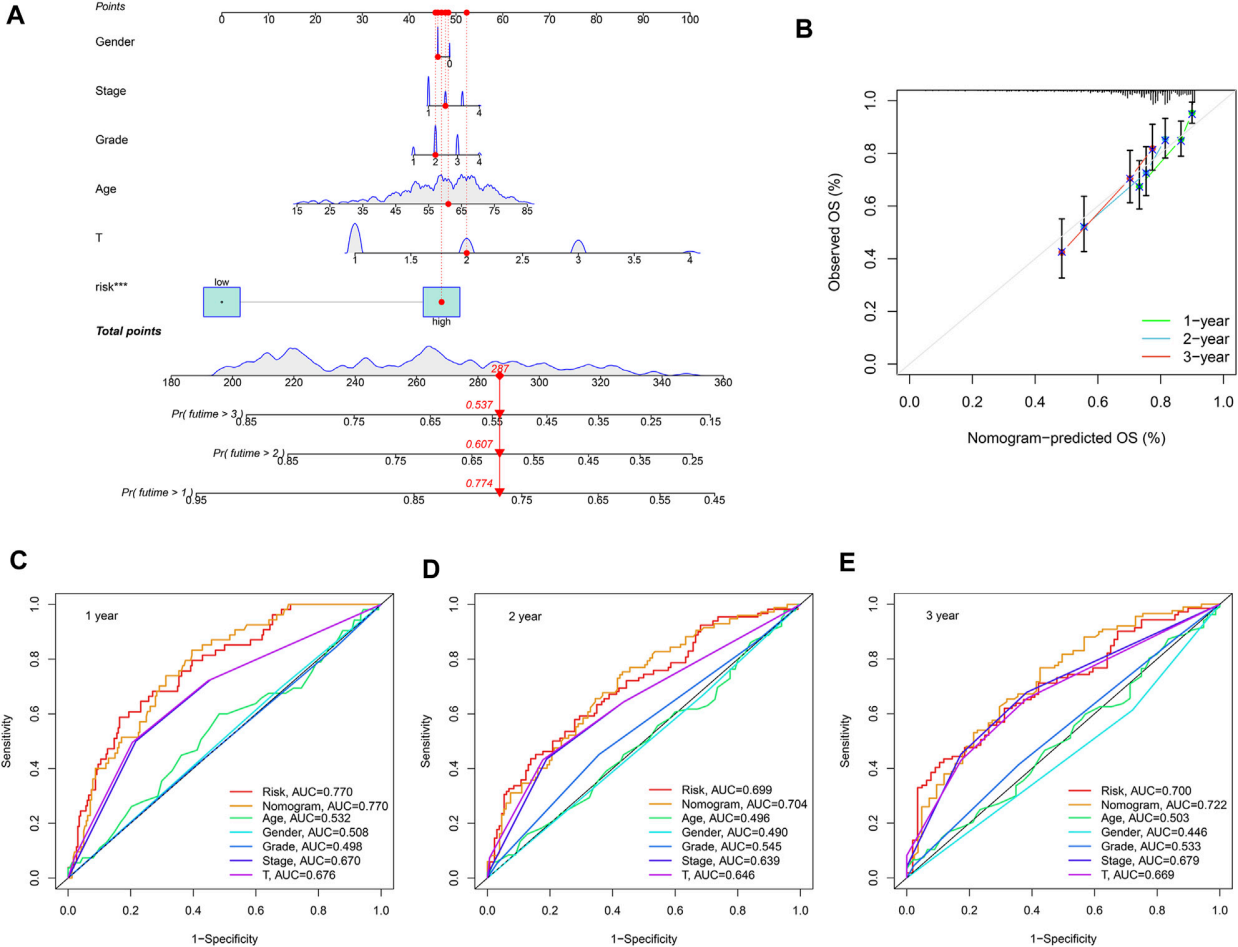
Construction of the nomogram. **(A)** The 1-, 2- and 3-years survival rates predicted by Nomogram were 0.774, 0.607, and 0.537, respectively. **(B)** The Calibration curve showed that the survival rate predicted by nomogram is consistent. **(C–E)** multiple ROC results showed that risk score and the nomogram were better than Age, Gender, Grade, Stage, and T Stage in predicting HCC patients’ survival, which reflects the robustness of risk score and nomogram.

Since the previous survival analysis was performed between groups, we next evaluated the SLC-related signature in the prognostic assessment of a single sample. We randomly selected patient TCGA-K7-AAU7 from the TCGA database to construct the nomogram, and the results showed the predicted 1, 2 and 3-years survival rates of the patient to be 0.774, 0.607, and 0.537, respectively (Figure 8A). Then, the calibration curve was used to evaluate the accuracy of the nomogram, with the results showing that the nomogram had a high degree of accuracy in predicting the survival rates of HCC patients (Figure 8B). Finally, multiple ROC results showed that the risk score and the nomogram were more accurate than age, gender, grade, stage and T stage in predicting HCC patients’ survival rates, thus confirming the robustness of the risk score and the nomogram (Figure 8C).

### PCR was Performed to Verify the Expression of the Key Gene SLC7A11

**FIGURE 9:**
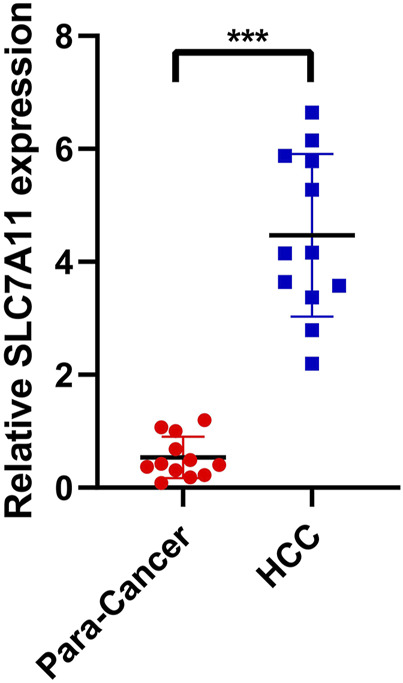
PCR was performed to verify the expression of the key gene SLC7A11. SLC7A11 was significantly overexpressed in HCC compared with adjacent normal tissues.

To verify the expression of SLC7A11 in hepatocellular carcinoma, PCR assay was performed. A total of 12 pairs of hepatocellular carcinoma samples and adjacent normal tissues were analyzed. The ethics committee of Fuyang Hospital affiliated to Anhui Medical University approved the study. Results showed that SLC7A11 was significantly overexpressed in HCC compared with adjacent normal tissues (Figure 9, ***p < 0.001).

## Discussion

The solute carrier (SLC) transporter family mediates the transmembrane transport of a variety of metabolic substrates which are essential for maintaining normal cell function (Song et al., 2020). In the process of tumorigenesis, the unlimited proliferation of cells means that their metabolic rates are high, thus requiring a large number of substrates and products to enter or be transported out of cells (Deng et al., 2020). Therefore, tumorigenesis is inevitably accompanied by changes in the expression of membrane transporters, especially SLC transporters (Xie et al., 2021). The liver is the largest metabolic and immunological organ in the body; thus, the relationship between the occurrence of hepatocellular carcinoma and SLC transporters is inevitably closer than for other cancer types (Fang et al., 2021). It is of great value to explore the role of SLC transporters in hepatocellular carcinoma.

In this study, we comprehensively assessed the value of 456 SLC transporters in HCC. The SLC transporter-related prognostic signature of HCC was constructed by LASSO regression and Cox regression. This provides a risk score formula for HCC patients. Based on the median risk value, HCC patients can be divided into a high-risk and a low-risk group, with the high-risk group having a significantly poorer prognosis. Subsequently, we explored the differences between the two groups in terms of immune infiltration and immune checkpoint in order to evaluate the role of the prognostic signature in guiding immunotherapy. The results revealed differences in the levels of immune cell infiltration and immune checkpoint gene expression between high-risk and low-risk groups. These findings can provide a reference for the immunotherapy treatment of HCC. Among the nine SLC transporters in the signature, we identified the hub gene SLC7A11 through interaction network analysis. This gene was found to be a strong biomarker for HCC.

At present, the incidence of hepatocellular carcinoma is still increasing (Hartke et al., 2017). Although viral hepatitis remains the primary cause of HCC, non-alcoholic steatohepatitis (NASH) is emerging as the fastest-growing risk factor (Luci et al., 2020). HCC is a highly heterogeneous tumor; this is due to a combination of multiple pathogenic factors (Kim et al., 2017). In the case of early HCC, surgical resection, local radiofrequency ablation, transarterial embolization (TARE) and liver transplantation have all shown preliminary efficacy (Wang and Wei, 2020). However, current treatment options for advanced HCC are still limited. Although current clinical trials have shown that sorafenib combined with immunotherapy (such as immune checkpoint inhibitors) yields good initial results in the treatment of unresectable liver cancer, the drug response rate remains low (Ruf et al., 2021). Moreover, we still lack a specific model for evaluating the prognosis and immunotherapy response of HCC. Our study can provide a specific SLC transporter-based risk score formula for the prognosis assessment of HCC patients, which will undoubtedly benefit the diagnosis and treatment of HCC patients.

The hub gene which we identified—SLC7A11—has been preliminarily discussed by several studies in relation to its significance in HCC. Wang et al. found that the interaction between SLC7A11 and DAZAP1 is closely related to the progression of HCC and is a potential target for the treatment of the disease (Wang et al., 2021). Lyu et al. found that in HCC, cirC0097009 regulates ferroptosis in HCC cells by regulating SLC7A11 (Lyu et al., 2021). Huang et al. found that ABCC5 downregulates the ferroptosis process of HCC cells by stabilizing the SLC7A11 protein (Huang et al., 2021). Thus, SLC7A11 plays an important role in HCC. Our study found that SLC7A11 is a hub gene in the signature of nine SLC-related genes. SLC7A11 can also serve as a robust marker for the prognostic stratification of HCC. In addition, SLC7A11 was associated with the infiltration of a variety of immune cells. Therefore, the value of SLC7A11 in the diagnosis and treatment of HCC warrants further exploration in the future.

The signature that we constructed contains a number of other genes with similar significance for the pathophysiology of hepatocellular carcinoma. They mediate the transport of a group of solutes, among them amino acids, zinc and prostaglandin. Chen et al. found that mutations of SLC25A19 protein in nerve cells resulted in impaired thiamine pyrophosphate (TPP) transport activity (Chen et al., 2021). These findings suggest that SLC25A19 may play an important role in mediating the transmembrane transport of TPP. The significance of SLC16A3 in cancer has been preliminarily discussed, in terms of its role of mediating the transmembrane transport of various substances such as fatty acids and lactic acid (Choi et al., 2019). Our previous study also revealed that SLC16A3 is highly expressed in lung adenocarcinoma and is associated with poor prognosis and immune cell infiltration (Xue et al., 2021). SLCO2A1 mediates the transmembrane transport of thromboxane and prostaglandin (Nakanishi et al., 2021). SLCO2A1 has been revealed to play a key role in many pathophysiological processes and is considered a potential pharmacological target for the treatment of diabetic foot ulcers. Zhu et al. revealed that SLCO2A1 is a promising therapeutic target for lung cancer in that it mediates the invasion and apoptosis of lung cancer cells through the PI3K/AKT/mTOR pathway (Zhu et al., 2015). SLC36A1 mediates the transmembrane transport of amino acids, and Yoshida et al. found a correlation between SLC36A1 and drug resistance in melanoma (Yoshida et al., 2019). SLC39A10 is a zinc transporter (Ma et al., 2021). Ma et al. found that SLC39A10 is upregulated in HCC cells and is associated with poor prognosis and immune cell infiltration (Ma et al., 2021). SLC30A3 has also been found to be a zinc transporter, and Zhang et al. discovered that SLC30A3 is a potential target for the treatment of glioma (Zhang et al., 2021). Studies on the roles of SLC38A6 and SLC17A8 in tumors are not clear. In summary, our study integrates a number of different SLC transporters which mediate the transport of different substrates, thus forming a substrate transport network. The signature we constructed can not only evaluate the prognosis of HCC patients but also provide a reference for the metabolic characteristics of HCC.

## Conclusion

Our study provides a risk score signature for hepatocellular carcinoma based on nine SLC genes. HCC patients can be accurately grouped by calculating their risk scores. We also discovered a robust biomarker of HCC: SLC7A11. Our study can provide a valuable reference for the diagnosis and treatment of HCC.

## Data Availability

The original contributions presented in the study are included in the article/Supplementary Material, further inquiries can be directed to the corresponding authors.

## Abbreviations

SLC, solute carrier; SLC7A11, solute carrier family 7 member 11; SLC27A5, solute carrier family 27 member 5; TCGA, The Cancer Genome Atlas; ICGC, International Cancer Genome Consortium; UCSC, University of California Santa Cruz; ROC, Receiver Operating Characteristic Curve; PCA, principal component analysis; T-SNE, t-distributed stochastic neighbor embedding; SLC25A19, solute carrier family 25 member 19; DAZAP1, DAZ associated protein 1; ABCC5, ATP binding cassette subfamily C member 5.
