# Supplementary material for: Transcriptome Analysis of Solute Carrier-Associated Genes in Hepatocellular Carcinoma: Friend or Foe?
Source: Front Genet. 2022 Mar 23;13:856393. doi: 10.3389/fgene.2022.856393 (PMC8984160; doi:10.3389/fgene.2022.856393)
Supplement: Supplementary file 2 [file Table1.docx]

**Table S1: Primer sequence used in this study**

| β-actin (Forward) | TCAAGATCATTGCTCCTCCTGAG |
| --- | --- |
| β-actin (Reverse) | ACATCTGCTGGAAGGTGGACA |
| SLC7A11(Forward) | CTTCATGGTTGCCCTTTCCC |
| SLC7A11(Reverse) | TAATGTTCTGGTTATTTTCTCCGAC |
